# Prescribing ANtiDepressants Appropriately (PANDA): a cluster randomized controlled trial in primary care

**DOI:** 10.1186/1471-2296-14-6

**Published:** 2013-01-08

**Authors:** Esther Muskens, Rhona Eveleigh, Peter Lucassen, Chris van Weel, Jan Spijker, Peter Verhaak, Anne Speckens, Richard Oude Voshaar

**Affiliations:** 1 Department of Primary and Community Care, Radboud University Nijmegen Medical Centre, Nijmegen, The Netherlands; 2 Trimbos Institute, Utrecht, The Netherlands; 3 Behavorial Science institute, Radboud University Nijmegen, Nijmegen, The Netherlands; 4 Department of Primary Care, University Medical Center Groningen, University of Groningen, Groningen, The Netherlands; 5 Netherlands Institute for health service research (NIVEL), Utrecht, The Netherlands; 6 Department of Psychiatry, Radboud University Nijmegen Medical Centre, Nijmegen, The Netherlands; 7 University Center for Psychiatry & Interdisciplinary Center for Psychopathology of Emotion regulation (ICPE), University Medical Center Groningen, University of Groningen, Groningen, The Netherlands

**Keywords:** Depression, Anxiety, Composite International Diagnostic Interview (CIDI), Randomized controlled trial, General practice, Depressive disorder, Anxiety disorders

## Abstract

**Background:**

Inappropriate use of antidepressants (AD), defined as either continuation in the absence of a proper indication or continuation despite the lack of therapeutic efficacy, applies to approximately half of all long term AD users.

**Methods/design:**

We have designed a cluster randomized controlled clinical trial to assess the (cost-) effectiveness of an antidepressant cessation advice in the absence of a proper indication for maintenance treatment with antidepressants in primary care.

We will select all patients using antidepressants for over 9 months from 45 general practices. Patients will be diagnosed using the Composite International Diagnostic Interview (CIDI) version 3.0, extended with questions about the psychiatric history and previous treatment strategies. General practices will be randomized to either the intervention or the control group. In case of overtreatment, defined as the absence of a proper indication according to current guidelines, a cessation advice is given to the general practitioner. In the control groups no specific information is given. The primary outcome measure will be the proportion of patients that successfully discontinue their antidepressants at one-year follow-up. Secondary outcomes are dimensional measures of psychopathology and costs.

**Discussion:**

This study protocol provides a detailed overview of the design of the trial. Study results will be of importance for refining current guidelines. If the intervention is effective it can be used in managed care programs.

**Trial registration:**

NTR2032

## Background

Depressive- and anxiety disorders are among the most prevalent disorders, with lifetime prevalence rates of 19% for both [1]. Most patients with depressive- and anxiety disorders are treated in general practice. In the last decade of the 20th century, prescription rates for antidepressants have increased 4 to 10 times in general practice [2,3]. Currently, 2.1–2.5% of patients treated in primary care receive antidepressants for 9 months or longer, i.e. 50–60 patients per average Dutch general practice with 2350 patients [4]. The appropriateness of long-term antidepressant usage is a matter of debate [5]. From a patient perspective, inappropriate use of antidepressants has serious consequences for safety, wellbeing and daily functioning [5]. Also there are negative side effects such as sexual disorder, emotional flattening, interaction with other drugs and sedation [6]. From an economic perspective, inappropriate use of antidepressants is expensive considering the high costs of modern antidepressant drugs in the case of overtreatment and the high costs for productivity loss due to depressive and anxiety disorders in the case of undertreatment. Although the Dutch NEMESIS data show that half of the identified antidepressant drug users still suffered from a depressive or anxiety disorder [5] and are thus in need of subsequent treatment steps we considered this type of inappropriate treatment beyond the scope of the study reported here.

Current guidelines advise to continue treatment with antidepressants for 6 months after remission for a first or second depressive episode or a successfully treated anxiety disorder, which means a total treatment duration of approximately 9 months [7,8]. Overtreatment is therefore defined as the continued prescription of antidepressants without an appropriate indication at start or continued prescription during more than 6 months after remission of the index disorder. There is evidence of overtreatment in primary care. Antidepressants are often initiated during the first consultation with the general practitioner (GP) about emotional symptoms [9]. Moreover, up to 80% of the users receive antidepressants for mild to moderate depressions [4,10-12], while 60% of these depressions remit spontaneously within 6 months [5]. The Netherlands Mental Health Survey and Incidence Study, has shown that about half of the antidepressant drug users in the community did not meet the criteria for a depressive or anxiety disorder in the past six months. Similar results were found in chart-review studies, showing that GPs had not registered a psychiatric diagnosis in nearly 40% of patients receiving prescriptions for antidepressant drugs. [3,4,13] Furthermore, 10–15% of long-term antidepressant drug users continue usage after remission without trying to discontinue it.

### Aims of the study

The aim of this study is to reduce ‘overtreatment’, i.e. long-term use of antidepressants (>9 months) in the absence of an indication for maintenance treatment with antidepressants according to current guidelines. We will evaluate the (cost-)effectiveness of a cessation advice to the general practitioner based on a detailed patient assessment in general practice.

### Study hypotheses

The main hypothesis is that the intervention will lead to a higher reduction in antidepressant usage as well as lower (in)direct costs compared to the control condition.

A second hypothesis is that these gains will be achieved without deterioration of psychological functioning.

## Methods/design

### Design of the trial

**Figure 1:**
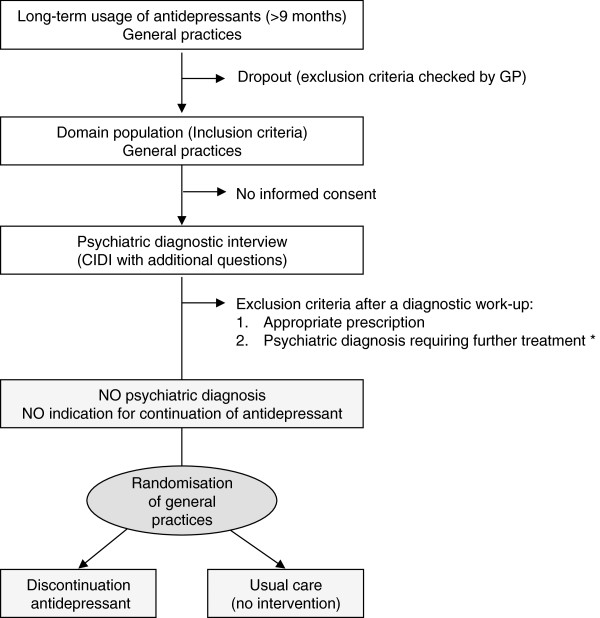
**Study flowchart cluster randomisation.** * Informed consent will be asked for a randomised controlled trial conducted in tandem to improve ‘undertreatment’ in long-term antidepressant users. Abbreviations: GP, general practitioner; CIDI, Composite International Diagnostic Interview.

This study consists of a randomised controlled parallel-group trial, which will be conducted in general practice (see Figure 1 for an overview). Patients using antidepressants for at least nine months or longer are eligible for participation. Potentially eligible patients will be identified within the computerised prescription databases of the participating general practices. The GP will check the exclusion criteria (see below) for the patients on these generated lists. After a structured psychiatric interview using the Composite International Diagnostic Interview (CIDI) 3.0 [14] with added detailed questions about psychiatric history and treatment, patients meeting the inclusion criteria will included in the trial. In case of undertreatment, i.e. the presence of a psychiatric diagnosis despite long-term antidepressant usage, patients will be offered to participate in a second trial that will be conducted in tandem (see trial registration database: NTR2032). As we failed to recruit sufficient patients for this second trial, no further details will be given here.

The primary outcome measure is successful discontinuation of antidepressant drug use, defined as no use of antidepressants during the 6–12 month follow-up in the absence of a psychiatric disorder. At the 12-month follow-up, the CIDI 3.0 will be re-administered by an interviewer blind for baseline results. Secondary outcome measures include a detailed set of dimensional measures of psychopathology, quality of life and costs (see below) administered at baseline and at 3, 6, 9, and 12 months follow-up (see below).

### Setting

The study will be conducted in general practice. Between February 2010 and January 2012, a total of 56 general practices were contacted for participation of which 45 practices (response rate 80.3%) have actually participated in the study: 31 practices from the Primary Care Network of the Radboud University Nijmegen Medical Centre and 14 practices not connected with this network.

### Patients

#### Eligibility assessment

Patient recruitment took place between May 2010 and May 2012. General practitioners systematically identified all users of antidepressants using the prescription records of their electronic medical records (supported by our research team). However, GPs were able to exclude long-term users a priori based on the exclusion criteria (known to them) or specific reasons that had to be specified per patient. All remaining patients using antidepressants for over nine months were considered eligible and received written information about the study. Those who consented to participate were contacted by the researcher for further information and screened once more on the in- en exclusion criteria. Patients who still met the criteria received a formal appointment for structured psychiatric interview by telephone using the Composite International Diagnostic Interview, version 3.0 (CIDI 3.0).

#### Inclusion criteria

1. Having received prescriptions for antidepressants for at least nine months in an amount sufficient for at least 270 days of use according to the prescribed dosage extracted from the electronic prescription records. Except for MAO-inhibitors, all types of antidepressants are included in this study. We do not include patients who receive MAO-inhibitors because these drugs can only be prescribed by psychiatrists in the Netherlands.

2. Having given written informed consent before the date of the psychiatric interview.

### Exclusion criteria

1. Appropriate use of long-term antidepressants according to the Dutch guidelines for depressive and anxiety disorders, i.e. a) a history of recurrent depression with 3 or more episodes and/or a recurrent psychiatric disorder with at least two relapses after antidepressant-discontinuation.

2. Presence of a current psychiatric diagnosis for which antidepressants may be effective, i.e. depressive disorder, dysthymia, generalised anxiety disorder, panic disorder, or social phobia).

3. Current treatment in a psychiatric setting

4. History of psychosis, bipolar disorder, or obsessive compulsive disorder

5. Current diagnosis of substance use disorder;

6. Non-psychiatric indication for long-term antidepressant usage, e.g. neuropathic pain

7. Inability to perform the necessary assessment due to a hearing impairment (telephonic interview) and/or not understanding Dutch language (telephonic interview and survey).

A total number of 146 patients have been included. The one-year follow up interviews have started in May 2011 and are still going on, expectedly till May 2013.

### Randomization

To prevent contamination between intervention and control group a cluster randomisation is performed with the general practice as the unit of clustering. Random assignment was ensured by picking a sealed envelope with intervention or control group after patient recruitment had been concluded in that particular practice. The cessation advices will only be sent to the GPs from practices allocated to the intervention group. GPs from practices in the control group are asked to continue their usual care, as if they were not participating in this trial. Interviewers who conducted the baseline and follow-up interviews as well as the psychiatrist and general practitioner who judged the indication of maintenance treatment will remain blinded throughout the trial.

### Intervention

The intervention implies the discontinuation of antidepressant use, following the recommendations in the Dutch multidisciplinary guidelines for depressive and anxiety disorders [15]. These are similar to those in the British NICE guidelines, recommending strict indications for the initiation, continuation and discontinuation of antidepressants [7]. Although in a recent meta-analysis patients with a single depressive episode showed no difference in relapse rates between abrupt and gradual antidepressant discontinuation [16], we advised a gradual tapering program for the following reasons: 1) abrupt discontinuation may trigger a relapse in patients who suffered from an anxiety disorder or a recurrent depressive disorder at the time of initiating the antidepressant [16], and 2) discontinuation symptoms occur more frequently in patients who abruptly discontinue their antidepressants than in patients whose treatment is gradually tapered [17,18]. The general prac-titioner receives a letter stating that the patient does not meet the criteria for a depressive or anxiety disorder in the past six months. In addition, he or she receives an information sheet with current guidelines on antidepressant tapering and information about the discontinuation syndrome [19], including a detailed scheme for tapering for each patient (see Table 1). Duration of tapering was primarily based on the dosage and the half-life of the different antidepressants. No treatment restrictions are imposed on GP or patient in case of relapse or onset of a new psychiatric disorder after discontinuation.

**Table 1:** Applied schemes for tapering long-term antidepressant usage in primary care

**Antidepressant**	**AD tapering scheme (steps per 2 weeks, dose in mg/day)**				
	**Start dose**	**Step 1**	**Step 2**	**Step 3**	**Step 4**
**TCA**					
· Amitriptyline	> 150	150	100	50	25
· Imipramine	> 150	150	100	50	25
· Nortriptyline	> 150	150	100	50	25
· Clomipramine	> 150	150	100	50	25
**SSRI**					
· Fluoxetine	> 60	40	30	20	10
· Paroxetine	> 40	40	30	20	10
· Sertraline	> 150	150	100	50	25
· Citalopram	> 40	40	30	20	10
· Escitalopram	> 20	20	15	10	5
· Fluvoxamine	> 150	150	100	50	25
**Other antidepressants**					
· Mirtazapine	> 45	45	30	15	-
· Duloxetine	> 120	120	90	60	30
· Venlafaxine	> 150	150	112.5	75	37.5
· Trazodon	> 150	150	100	50	-

A psychiatrist (RCOV) and a general practitioner (PL) will compare the diagnostics and treatment history of patients who underwent a full baseline examination with current guidelines independently. The decision for a cessation advise will be based on the Dutch Multidisciplinary Guidelines for the treatment of depressive disorder and of anxiety disorders [15]. These Multidisciplinary Guidelines provide detailed information and a treatment algorithm for all depressive and anxiety disorders. In a case of incongruent advices the psychiatrist and the general practitioner will discuss the case until they reach consensus. Incongruence between the GP and psychiatrist will be reported as percentage disagreement. When they are unable to reach consensus, advice will be asked from another couple consisting of a GP (CvW) and a psychiatrist (AS).

To check the reliability of the proposals by the GP and psychiatrist, we provided another GP (CvW) and psychiatrist (AS) with 10 randomly selected case vignettes from included patients. Comparing these judgments, there was a 100% agreement.

### Control condition

The control condition will consist of usual care and do not impose restrictions on GPs to deliver care or to refer to specialised mental health care, including the continuation or discontinuation of psychotropic drugs. Since baseline psychiatric diagnostics will not be disclosed for patients who have given informed consent in a control practice (also those with appropriate use or undertreatment), we expect continuation of antidepressant drug treatment in most cases [13].

### Assessments

Eligible patients who consent for participation will receive a psychiatric interview by telephone, using the depression and anxiety part of the Composite International Diagnostic Interview (CIDI) [20,21] as well as the sections on social phobia, bipolar disorder, generalized anxiety disorder, neurasthenia, specific phobia and obsessive compulsive disorder. The CIDI is a structured and fully standardized psychiatric interview for diagnosing mental disorders according to ICD and DSM-IV criteria.

The CIDI interview can be done by trained laymen, and thus imposes no restrictions on the interviewers. Furthermore, telephonic administration of the CIDI has been demonstrated feasible and reliably [20,21]. To enable the preparation of treatment proposal, the CIDI interview was extended with detailed questions about previously used psychotropic drugs (duration and dosages) and psychosocial therapies. In case of psychotherapy, predefined questions were asked to discern between cognitive-behavioral interventions, interpersonal therapy and/or structured/supportive therapy. In addition, demographic variables, the use of psychoactive substances (nicotine, alcohol, and drugs), screening of post-traumatic stress disorder, and the presence of chronic somatic disorders will be recorded at the baseline interview. The CIDI 3.0 interview will be repeated after one-year follow-up.

Subsequently, all patients will fill out a set of self-report questionnaires at baseline and at 3, 6, 9 and 12 months follow-up (see secondary outcome measures below).

Personality characteristics and the quality of the patient-physician relationship have been suggested to affect treatment outcome of common mental disorders in primary care. Therefore, these characteristics will be examined additionally at baseline by administration of the NEO-Five Factor Inventory (NEO-FFI) [22] and the Patient Doctor Relation questionnaire (PDRQ) [23].

### Primary outcome measures

The primary outcome is the proportion of participants who successfully discontinue their long-term antidepressive drug use. This is defined as having no antidepressant drug use within the last 6 months of the follow-up and the absence of a depressive or anxiety disorder during one-year follow-up as assessed with the CIDI 3.0. Use of antidepressants will be evaluated with questions during this second CIDI interview as well as with self-report questionnaires. In case of inconsistencies between both measures, the patient will be re-contacted and if necessary the GP will be contacted to check the GP prescription database. This latter solution is considered reliable as in the Netherlands all patients are linked to only one GP who collects all medical information for that patient.

### Secondary outcome measures

Secondary outcome measures are the severity of psychological symptoms, quality of life, costs and also the prevalence of discontinuation symptoms. We have only included self-report questionnaires that are validated in Dutch and have shown good to excellent psychometric properties.

The overall severity of psychological distress and global psychopathology will be based on the Brief Symptom Inventory (BSI-53) sum score. The BSI-53 is a shortened version of the Symptom Checklist (SCL) 90-item version [24] with similar psychometric characteristics and subscales, but less patient burden [25].

For more detailed evaluation, we also included disorder specific instruments:

– The Centre for Epidemiological Studies Depression Scale (CES-D) for measuring the severity depressive symptoms [26];

– The Penn State Worry Questionnaire (PSWQ) for assessing the frequency and severity of symptoms of worrying [27];

– The Panic and Agoraphobic Scale (PAS) for measuring the severity of illness in patients with panic disorder [28];

– The Fear of Negative Evaluation Scale (FNES) for assessing expectations and distress associated with negative evaluations by others [29];

The EuroQol-5D (EQ-5D) offers the possibility to evaluate effects of quality of life, although this questionnaire is primarily included to enable the economic analyses by providing a utility score [30-33]. The EQ-5D has previously been validated and has been applied successfully in studies of depressive and anxiety disorders.

Costs will be measured by the Trimbos/iMTA questionnaire for Costs associated with Psychiatric Illness (TiC-P) [34].

Finally, the prevalence and severity of an antidepressant discontinuation syndrome will be measured in those who withdraw from medication using the Discontinuation-Emergent Signs and Symptoms (DESS) Scale [35]. This latter scale, however, has not been validated in Dutch.

### Power

A senior academic statistician using SAS POWER procedure performed a prospective sample size calculation. It aimed to determine a target sample size that would provide at least 85% power for two-tailed testing (at a type-1 error rate of 5%). Because our trial is cluster randomized, calculations to determine the minimum number of general practices is stricter than in a non-clustered trial. To account for this, we used an intra-class correlation (ICC) of 0.05.

Assumptions with respect to recruitment and outcome are difficult to estimate. We expect a 20% discontinuation rate for patients in the control group, and a 50% discontinuation rate in the intervention group. The 20% discontinuation rate is conservatively estimated (probably lower than 20%) based on the fact that in the Netherlands, the rate of spontaneous non-adherence to antidepressant drug therapy has been estimated at 25% within the first 6 months [36]. This rate is expected to decline gradually as the treatment time elapses (as those patients with initial side-effects have already dropped out). The 50% discontinuation rate is based on the results of a primary care benzodiazepine discontinuation study of our group [37,38]. This is also considered conservative, as in contrast to benzodiazepines, psychological dependency does not play a major role in long-term use of antidepressants.

The recruitment rate was originally based on a small qualitative pilot study assuming that one average general practice would enable to include 6 patients in the trial. Assuming a dropout rate of 25%, a total of 20 practices (160 patients) had to be recruited. As the number of patients recruited per practice was lower than expected, we recruited more practices for participation in the study. A total of 45 practices finally participated in the study, which resulted in the recruitment of 146 patients. Based on our a priori assumptions of the success rates and drop out rates, we will have over 85% power.

## Data analysis and treatment effect

### Descriptive statistics

The trial has a binary primary outcome, i.e. successful discontinuation yes or no, and dimensional secondary outcome parameters. In order to check for baseline differences between the two groups, a series of univariate analyses (t-tests, chi-square tests, Mann–Whitney tests) on psychiatric status and demographic variables will be performed.

### Multilevel analysis

Multilevel analyses will be used to account for the hierarchical structure of the data (i.e. patients nested within practices). Covariates (see above) will be included if they show a relationship with the outcome. The secondary outcome measures (continuous variables) will be analysed by using the mixed procedure in SAS. Firstly, a general comparison will be made based on the BSI-53 (general distress), which is applicable to all patients. Secondly, disease-specific instruments will be pooled after having determined the most relevant disease-specific questionnaire for each patient based on one’s primary diagnosis assessed with the CIDI at study entry (i.e. depressive disorder, panic disorder, generalized anxiety disorder or social phobia). The scores on the different questionnaires will be transformed into standardized t-scores, in order to pool these data in multilevel analysis for continuous variables. In case the CIDI at baseline will not identify any psychiatric disorder for which the antidepressant drug treatment has been initiated, the BSI-53 score will be taken.

All analyses will be performed on an intention-to-treat basis. Patients who dropped out will be classified as failure (for the primary outcome variables). For the secondary outcome measures, missing values will be imputed by multiple imputation techniques.

### Interim analysis

The investigators do not expect any serious adverse events that will require an interim analysis to make a deliberate consideration of terminating the study earlier than planned (approved by the institutional ethics committee). Due to the statistical characteristics of an interim analysis, the achieved power of this study would be unnecessarily reduced.

### Ethical aspects

The trial is registered before start of the study, and will be reported according to CONSORT guidelines. Our study is approved by the institutional ethics committee Nijmegen under registration number NL29718.091.09 and registered in the Netherlands Trial Register NTR2032.

Informed consent is obtained from the subjects before entering the study. Before patients give their consent, a detailed information package is sent to them, which provides the aims and characteristics of the study. All subjects are informed that participation in the study is voluntary and that they are allowed to withdraw from the study at any time.

## Discussion

We have described the study protocol of a cluster-randomised controlled clinical trial to evaluate the impact of cessation advice to the GP in order to reduce overtreatment with antidepressants in general practice. The primary outcome measure is the proportion of patients that successfully discontinued their antidepressants. Secondary outcomes include dimensional measures of psychopathology and (in)direct costs.

### Cluster randomization

We applied cluster randomization instead of individual or normal randomization for two reasons [39]. Firstly, randomization at the patient level would inevitably have led to contamination, as receiving cessation proposals for some, but not all patients included from an individual GP, would inevitably trigger him or her to rethink their strategy of control patients. During the trial, treatment given to control patients will probably be contaminated. Secondly, in our study the patients are nested in general practices and cannot be considered as statistically independent. Thus, when not taking into account the general practice as unit of cluster, this will inflate type I errors [40].

### Generalizability

The study is based on a pilot study, performed before the PANDA study started. In this pilot study there was a calculation made how many patients were using antidepressants for more than 9 months. We identified significantly fewer patients per general practice/general practitioner who participated in the study. This might limit the generalizability of our findings. Because of this finding we performed some recalculations of the power analysis (as described above). This is not necessarily problematic, since including fewer patients per practice will improve the power.

Furthermore, being a primary care study, we cannot generalise our results to those patients treated in psychiatric care. Overtreatment is probably also an issue in specialised mental health care, although to our knowledge exact figures are lacking. We excluded patients under current treatment in a psychiatric setting to prevent interfering with current specialised treatment. Nonetheless, most patients suffering from anxiety and/or depressive disorders are treated in primary care [41].

### Two trials in tandem

Classifying long-term users of antidepressants as being undertreated or overtreated requires a full psychiatric diagnostic work-up. The necessity and investment of this psychiatric work-up has led to the decision to conduct two trials in tandem using one recruitment strategy. Although available data suggested comparable proportions of patients being over- or undertreated, reality was hard-hearted. Recruitment rates in both trials were a little disappointing, but for the trial described in this paper we could include sufficient patients to achieve over 85% power based on a priori effect-sizes by recruiting more general practitioners to participate in our project. Nonetheless, we failed in recruiting sufficient patients for second trial aimed to improve undertreatment, i.e. the presence of psychiatric disorder despite the use of an antidepressant (which argues for further treatment steps in primary care). If not conducted in tandem, recruitment strategies for this second trial that was conducted parallel to the trial presented could have been elaborated, as done in many other randomised controlled trials facing recruitment problems. We thus have to conclude that our planned efficiency appeared to be inefficient.

### Treatment proposals

A psychiatrist and general practitioner made the cessation proposals independent of each other. The main decision for the present study was to determine whether patients received the antidepressants appropriately and if not, whether cessation should be recommended according to actual guidelines (in case of overtreatment) or further treatment steps should be taken to augment or change the treatment. Interestingly, the reliability of the treatment proposals was excellent, as shown by a 100% agreement between the results of the second psychiatric-GP couple on 10 randomly chosen patients.

### Clinical relevance of this trial (for update of guidelines)

This study aimed to answer questions on the (cost) effectiveness to taper antidepressant use in patients who have no recent diagnosis of depression and/or anxiety. These results will inevitably impact on the current guidelines. In case of non-discontinuation, more emphasis should be paid on strategies to discontinue antidepressants in primary care. In case of high relapse rates after discontinuation, guidelines should be adapted regarding the duration of maintenance treatment.

## Competing interests

The authors declare that they have no competing interests.

## Authors’ contributions

RCOV and PL designed the study and received funding for the study. RE and EM, supervised by RCOV, PL and AS, are responsible for conducting the trial (recruiting GPs, data-collection and analyzing the data of the trials). All other authors critically commented on the study design and present paper. All authors read and approved the final manuscript.

## Pre-publication history

The pre-publication history for this paper can be accessed here:

http://www.biomedcentral.com/1471-2296/14/6/prepub
